# A phantom study to contrast and compare polymer and gold fiducial markers in radiotherapy simulation imaging

**DOI:** 10.1038/s41598-021-88300-w

**Published:** 2021-04-26

**Authors:** Daryl Lim Joon, Drew Smith, Mark Tacey, Michal Schneider, Benjamin Harris, Wee Loon Ong, Farshad Foroudi, Trish Jenkins, Morikatsu Wada, Michael Chao, Kym Rykers, Vincent Khoo

**Affiliations:** 1 Department of Radiation Oncology, Newton-John Cancer Centre, Heidelberg, Olivia 3084 Australia; 2 Department of Medical Imaging and Radiation Sciences, Monash University, Clayton, 3800 Australia; 3 Royal Marsden NHS Foundation Trust, London, SW3 6JJ UK

**Keywords:** Oncology, Urology

## Abstract

To assess visibility and artifact characteristics of polymer fiducials compared to standard gold fiducials for radiotherapy CT and MRI simulation. Three gold and three polymer fiducials were inserted into a CT and MRI tissue-equivalent phantom that approximated the prostate cancer radiotherapy configuration. The phantom and fiducials were imaged on CT and MRI. Images were assessed in terms of fiducial visibility and artifact. ImageJ was employed to quantify the pixel gray-scale of each fiducial and artifact. Fiducial gray-scale histograms and profiles were generated for analysis. Objective measurements of the contrast-to-noise ratio (CNR), signal-to-noise ratio (SNR), and artifact index (AI) were calculated. The CT images showed that the gold fiducials are visually brighter, with greater contrast than the polymer. The higher peak values illustrate this in the line profiles. However, they produce bright radiating and dark shadowing artifacts. This is depicted by the greater width of line profiles and the disruption of phantom area profiles. Quantitatively this results in greater percentile ranges of the histograms. Furthermore, for CT, gold had a higher CNR than polymer, relative to the phantom. However, the gold CNR and SNR were degraded by the greater artifact and thus AI. Both fiducials were visible on MRI and had similar histograms and profiles that were also reflected in comparable CNR, SNR and AI. Polymer fiducials were well visualized in a phantom on CT and MR and produce less artifact than the gold fiducials. Polymer markers could enhance the quality and accuracy of radiotherapy co-registration and planning but require clinical confirmation.

## Introduction

The increasingly conformal dose distributions of intensity-modulated radiotherapy (IMRT) and volumetric arc therapy (VMAT) require a more precise target definition and treatment delivery to achieve an optimal therapeutic ratio. For contemporary prostate IMRT, the combination of fiducial alignment and soft tissue analysis is the most accurate and widely available image-guided radiotherapy (IGRT) method^1^. Importantly, IGRT with fiducial markers has been shown to improve treatment results^2^.

In addition, MRI plays a vital role in prostate radiotherapy contouring and increasingly in terms of verification on the MR linacs. Fiducial markers have been employed to assist in the co-registration of CT and MRI^3^. MRI, particularly T2 weighted MRI, can better visualize the normal anatomy and the tumor within the prostate for radiotherapy contouring^4–6^, thereby reducing inter-observer variation^7^. Thus, fiducials have potential CT and MRI roles for both image co-registration and X-ray-based verification, including electronic portal imaging devices (EPID) & cone-beam CT, and MRI verification on MRI linacs.

Gold fiducials are the most common fiducials used in prostate IMRT. Gold has a high Z value, making it radiopaque and highly visible with X-ray imaging. It is also safe to use with MRI and is biocompatible. However, the downside of the high Z value is a substantial artifact from the scattering of X-rays^8^. The artifact can interfere with the accurate fiducial definition leading to imprecise image guidance^9^. It can also obscure adjacent prostate and normal tissue anatomy leading to inaccurate target delineation. Alternative fiducial markers are available but are not well studied^1^.

Polymer fiducials (PolyMark, CIVCO) have been promoted as an alternative to gold because of reduced artifact on X-ray imaging and improved MRI visualization but have only undergone general evaluation^9,10^. They are regarded as safe, as they are used clinically in multiple countries having passed appropriate regulatory standards. Hence, before considering polymer fiducials for prostate cancer clinical use, we investigated the polymer fiducials in a CT and MR phantom study that approximated the prostate cancer scenario. The aim of the study was to assess the visibility and characterize the artifacts of the polymer fiducial compared to the standard gold fiducials on CT and MRI simulation images. If the results of this exploratory phantom study show a possible advantage for the polymer fiducials, then a clinical study using the methodological developments would be performed to assess whether they could replace the standard gold fiducials.

## Materials and methods

### Fiducial markers

**Figure 1:**
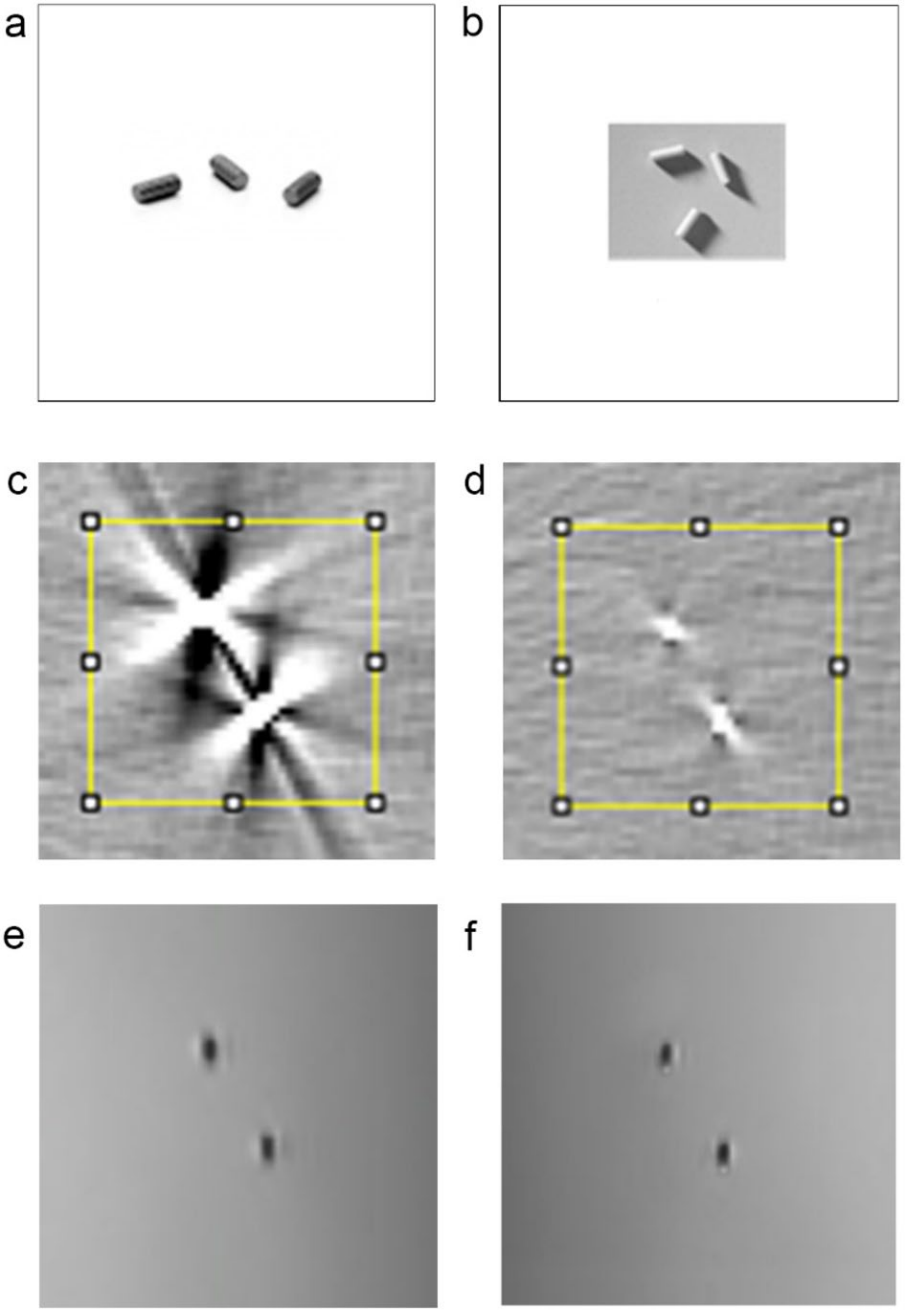
(**a**) Gold seeds, (**b**) Polymer seeds, (**c**) CT of gold seeds with the defined area for the gray-scale histogram, (**d**) CT of polymer seeds with the defined area for the gray-scale histograms, (**e**) MR Co-registered with CT of gold seeds and (**f**) MRI Co-registered with CT of polymer seeds.

Standard gold soft tissue fiducials measuring 0.9 mm × 3 mm and investigational PolyMark (polymer) fiducials markers measuring 1 mm × 3 mm and (CIVCO Medical Solutions, Kalona, Iowa, USA) were utilized for the study. (Fig. 1a and 1b).

### Phantom

**Figure 2:**
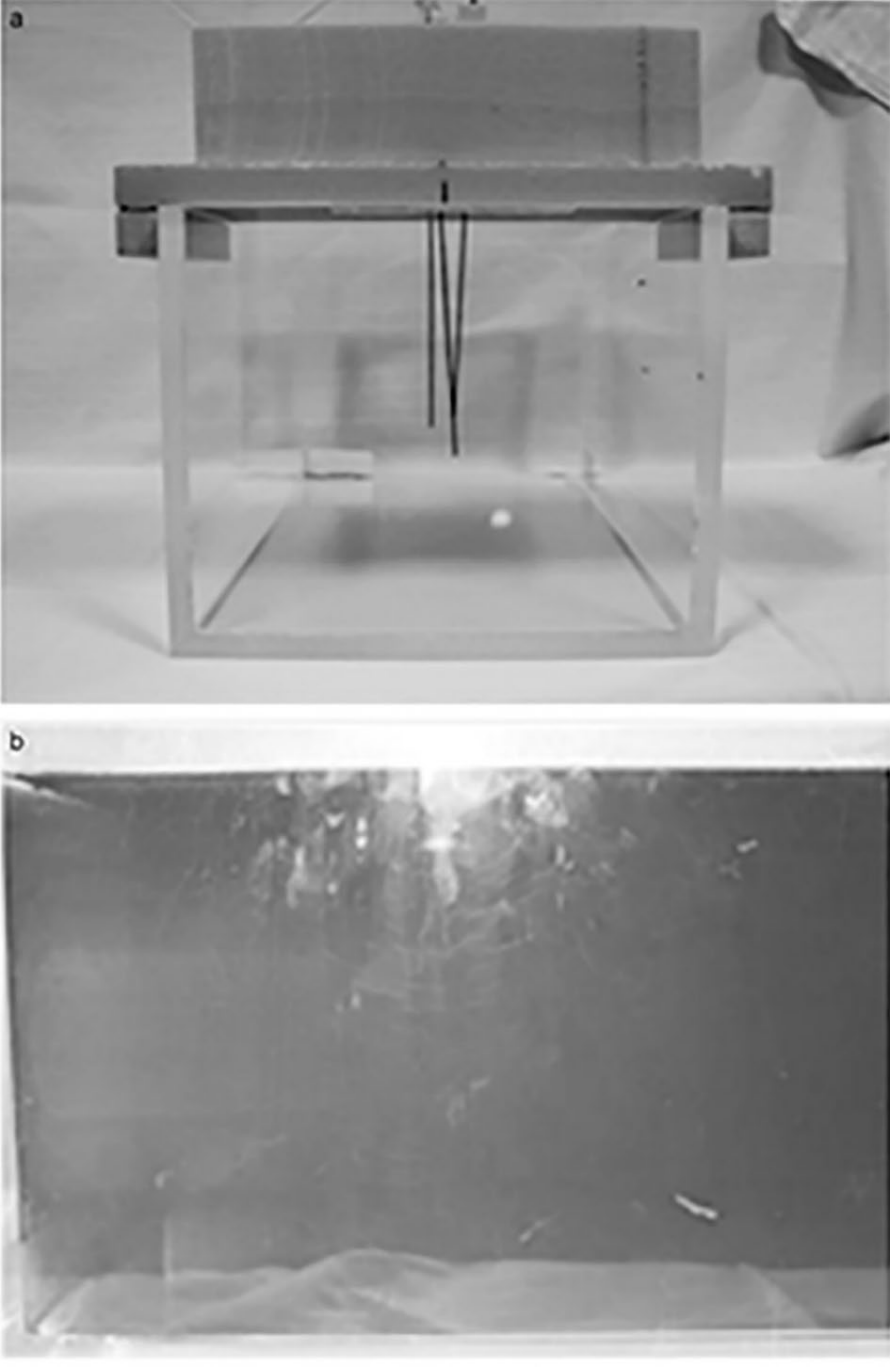
(**a**) Perspex box for the phantom with rigid foam plate to guide needles with gold seeds for reproducibility (**b**) phantom contained within perspex box.

A purpose-built, in-house, uniform, gelatine-based Phantom that was tissue equivalent in both CT and MRI was constructed specifically for the study as described in the following. A rectangular Perspex box, based on similar phantoms used for linear accelerator imaging quality assurance, was constructed to hold the phantom material (Fig. 2a). It measured 330 × 230 × 190 mm and was free from metallic components. A high-density foam grid template was created to ensure reproducibility and avoid positional inaccuracy between the test groups (Fig. 2a). This template positioned the gold and polymer fiducials precisely at either end of the phantom to simulate prostate cancer radiotherapy.

Following initial investigations, 200 ml samples were produced using both the 5% and 10% gelatine in addition to varying the diluent ratio of water to glycerol. Thus, three 5% gelatine mixes and three 10% gelatine mixes were created, using water to glycerol ratios of 1:1, 2:1, and 3:1 for each. The six samples were then imaged on CT and MRI.

The 10% gelatine in water to glycerol ratio of 2:1 was selected for the study because of its superior appearance with respect to tissue equivalence on CT and MRI. A sufficient volume of the 10% gelatine mixed with the 2:1 water:glycerol solution was created and poured into the Perspex box (Fig. 2b).

Once the mixture had set, the three gold and three polymer fiducial markers were placed within the phantom at mirrored positions using the template and native application apparatus needles (Sterile Placement Needle, 18GA ETW × 20 cm, CIVCO Medical Solutions, Kalona, Iowa, USA). The three gold fiducials and three polymer fiducials were inserted at either end of the phantom around the phantom’s long axis, 5 cm from the short phantom side, and centered around the mid-depth for both fiducial sets. The positioning of the three fiducials approximated that used in prostate cancer. The gold and polymer fiducials were positioned far enough from the sides and each other, so they did not interact with the Perspex or other fiducial set.

The phantom containing the polymer and gold fiducials was then imaged on CT and MRI in a rapid sequence according to the parameters below. This ensured scanning parameters and the phantom were uniform for all fiducial measurements on CT and MRI, as the phantom can dry out, changing its physical characteristics and appearance with time. Alternative phantom material would be required to acquire measurements over a more prolonged time.

### Imaging

The imaging parameters used for study CT and MRI are in accordance with those used for the clinical simulation for prostate cancer patients.

Simulation CT imaging was performed on a GE Lightspeed RT CT (Boston, Massachusetts, USA) with a 1.25 mm slice width, helical, 0.75 pitch, no gap, 512 × 512 axial resolution, and a 650 mm reconstruction diameter.

Simulation MRI was performed on a 1.5 T Siemens Magneto Avanto Syngo MR B17 (Siemens Healthcare, Erlangen, Germany). The MRI sequence utilized for this study was a high-resolution 3D T2-weighted scan with a voxel size of 1 mm, as it is used as the standard planning MRI scan at the center.

### Artifact measurement & analysis

Visual assessment, line, and area measurements were analyzed on DICOM transverse image files in a perpendicular plane corresponding to each fiducial markers center, i.e., three gold fiducials and three polymer fiducials.

ImageJ software (Rasband, W.S., ImageJ, U. S. National Institute of Health, Bethesda, Maryland, USA) was used to investigate and quantify the gold and polymer image artifacts fiducials. It was used to assess the individual pixel gray-scale values to generate line and surface profiles and histograms of gray-scale for MRI and CT across a region of interest.

The line profiles graphically highlight the gray-scale of the fiducials and artifacts within the phantom. Whereas the gray-scale histogram plots mainly represent the phantom and display thepPhantom’s disruption, that is mainly due to artifact, as the fiducials only occupy a relatively small number of pixels. The transverse CT and MRI images with the surface plots provide a visual comparison of the fiducials and the surrounding artifact terrain in the phantom, highlighting the artifact’s interaction with multiple fiducials. This relatively simple and effective method was developed to illustrate the fiducials’ similarities and differences in MR and CT.

The horizontal line profile plots were generated by centering a 50 mm and 20 mm line over each of the three gold and three polymer fiducial markers on CT and MR images, respectively. Longer lines were used with CT to incorporate the greater artifacts produced, particularly by the gold fiducials. The gray-scale reading from each pixel along the line was measured for each fiducial and normalized relative to the average phantom gray value, i.e., each pixel gray-scale was divided by the average gray-scale of the phantom material. Each pixel’s average value for both the three gold and three polymer fiducials was then plotted on the final line plots. The line plots were used to illustrate the change in the gray-scale relative to the distance across the phantom and fiducials. Line profile plots thus defined and highlighted the fiducial markers relative to the phantom.

Histograms and surface profiles were derived by centering a 50 mm square region of interest (ROI) over each fiducial marker on the center transverse slice for each respective seed. The raw gray-scale pixel data for each fiducial was exported to MS Excel for analysis. The average value for each pixel for both gold and polymer was used for the histograms and plots.

The histograms show the distribution of pixel number having a particular gray-scale value. The pixel gray-scale values were normalized relative to the background phantom gray value. Most of the gray-scale histogram represents the phantom as the fiducials occupy only a small number of pixels. A ratio of 1 represents the average phantom, whereas the spread of the histogram and slope relates to the artifact and its disruption of the phantom gray-scale. The relative gray-scale histograms were generated to highlight and contrast the artifact’s effect on the more uniform phantom gray-scale and indicate the amount of “true” gray-scale phantom seen.

Descriptive statistics were prepared to compare the relative gray-scale histograms for the polymer and gold fiducials for both the MRI and CT scans. Due to the non-normal distribution in the variation or range in voxel counts for the CT and MRI histograms, the relative gray values (artifact) attributable to each seed were measured as percentile ranges (1st to 99th, 5th to 95th, and interquartile range: 25th to 75th percentile).

To further quantify the fiducial visibility and artifact, objective measurements of the contrast-to-noise ratio (CNR), signal-to-noise ratio (SNR), and artifact index (AI) were calculated^11–13^. Two Backgrounds were used to compare the contrast (CNR) and signal (SNR), i.e. “Phantom” being distant from and not including the artifact, and the Region of Interest being the 50 mm square (“ROI”) containing the artifact. The parameters were calculated for the three polymer fiducial and three gold fiducials. The presented means (standard deviation, SD) relate to the CT number for CT scans and gray-scale for the MRI.

The CNR is an indicator of the relative image quality of the fiducial (i.e., visibility) being the ratio of contrast/noise. CNR equals the mean difference between the fiducial signal and the Background divided by the Background’s standard deviation, i.e. CNR = (mean Fiducial − mean Background)/(SD Background). The SNR compares the desired signal to the level of background noise, or signal/noise. The SNR was calculated from the quotient of the mean signal of the Background minus the fiducial and the SD where SNR = (Background mean excluding fiducial) / (Background SD excluding fiducial)^11,12^. This provides a measure of the impact of the artifact of the phantom’s image quality surrounding the fiducial. Both background noises and metal artifacts can increase the standard deviation (SD). When considering SD as an expression of the metal artifacts (AI), the influence of the Background (Background SD) should be subtracted. Therefore, the AI was calculated by AI = $\sqrt{({\mathrm{ROI\,SD \,but \,excluding \,fiducial})}^{2}-{\left(\mathrm{Background\, SD\, excluding\, fiducial}\right)}^{2}}$.

### Consent for publication

All authors consent to the publication.

## Results

### CT scan

The visual analysis showed that both the gold and polymer fiducials are bright and well seen on CT. However, the gold fiducials produce a greater amount of bright radiating and dark shadowing artifacts than the polymer seeds’ relatively minor artifact (Fig. 1c,d).

**Figure 3:**
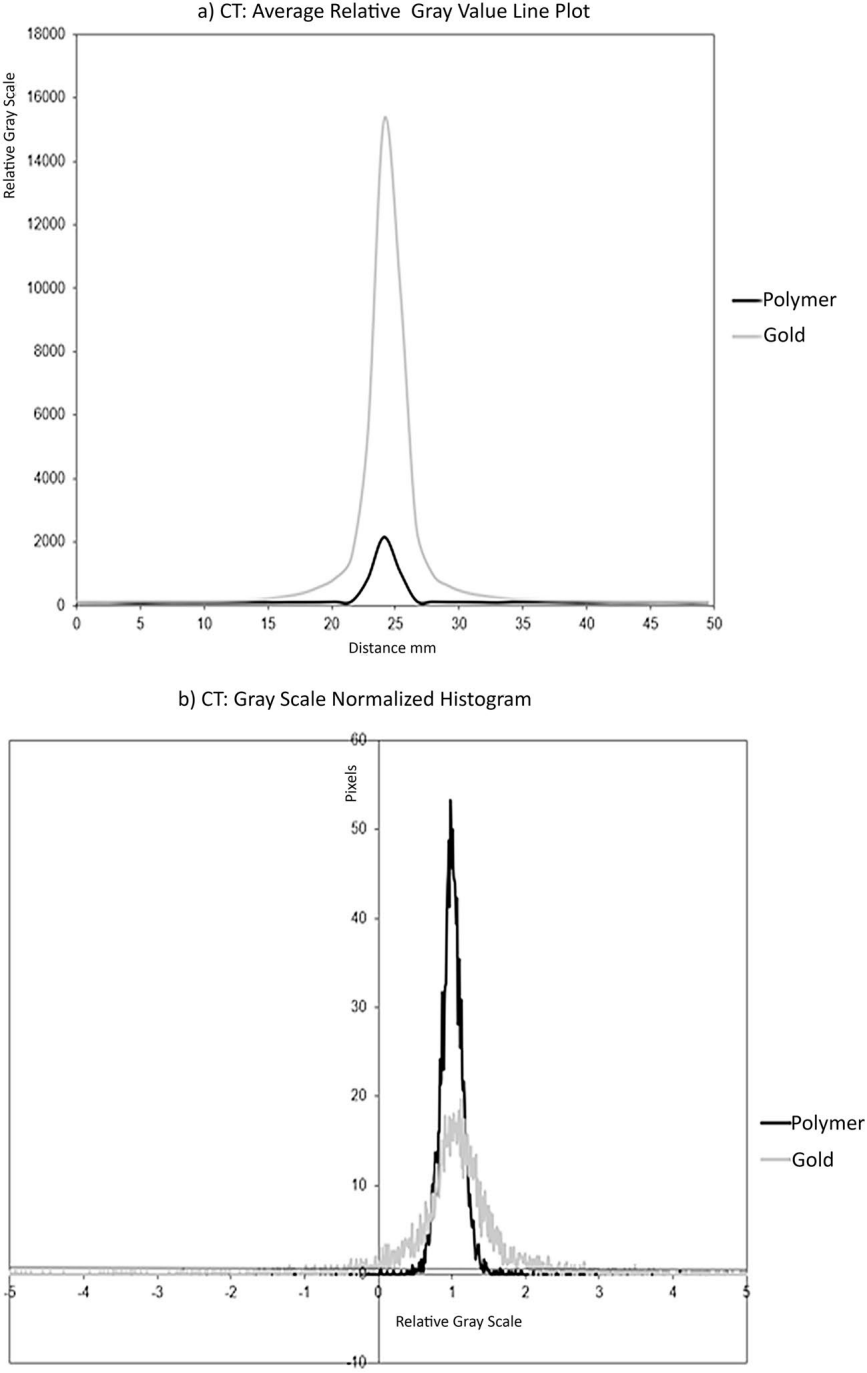
(**a**) line profile of average relative pixel gray values along a 50 mm horizontal line centered on each fiducial marker on CT, (**b**) CT histogram of average relative pixel gray values within a 50 × 50 mm square placed centered on each fiducial marker on CT, polymer and gold.

The CT horizontal gray-scale line profile was generated through the fiducial center (Fig. 3a). The gold fiducials have a higher peak (15,279 in relative gray-scale value) compared to polymer fiducials (2168), indicating they are brighter, with greater contrast relative to the phantom and the polymer seed. The gold fiducials also exhibit a broader base indicating a greater spread of gray values, with an approximate relative gray-scale range of 20 mm compared to approximately 5 mm for the polymer fiducials. Figure 3a is a graphical representation of the higher contrast and greater artifact of the gold fiducials compared to polymer fiducials.

The histogram of the normalized gray-scale versus pixel number (Fig. 3b) shows the effect of the fiducial and artifact on the phantom contained in the area of interest. The polymer histogram has a higher peak value around the centralized phantom gray value of 1, and the base was narrower (1st to 99th percentile: 0.6263 to 1.4848 for polymer fiducials compared to − 3.6061 to 6.6970 for gold fiducials (Table 1) (Fig. 3b) showing that there is less spread of gray-scale caused by the fiducial artifact and to a lesser extent the small fiducial itself. The greater amount of the phantom gray-scale being visible with the polymer fiducials is also illustrated by the smaller standard deviation surrounding the mean values and the narrower inter-quartile ranges surrounding the median and tighter percentile ranges (Table 1).

**Table 1:** A statistical comparison of the relative gray-scale histograms (where the phantom normal tissue gray-scale was normalized to 1) of the polymer versus gold fiducials, generated from a 50 mm square centered on the fiducial for CT and MRI imaging. The mean (SD standard deviation), median, and percentile ranges highlight and contrast the artifact’s effect on the more uniform phantom gray-scale.

Statistic	Polymer	Gold
**CT**		
Mean (SD)	1.0645 (1.5304)	1.1311 (2.1159)
Median (Inter-quartile Range)	1 (0.9091 to 1.0909)	1 (0.7374 to 1.2727)
5th to 95th percentile	0.7475 to 1.2525	− 0.3333 to 2.4343
1st to 99th percentile	0.6263 to 1.4848	− 3.6061 to 6.6970
**MRI**		
Mean (SD)	1.0369 (0.0505)	1.0390 (0.0455)
Median (Inter-quartile Range)	1.0009 (0.9943 to 1.0075)	1.0004 (0.9949 to 1.0077)
5th to 95th percentile	0.9812 to 1.0163	0.9839 to 1.0169
1st to 99th percentile	0.9636 to 1.0339	0.9748 to 1.0315

The mean (SD standard deviation), median, and percentile ranges highlight and contrast the artifact’s effect on the more uniform phantom gray-scale.

The mean (SD) of the CNR, SNR and AI of the polymer and gold fiducials are presented in Table 2. The gold fiducials had a much higher CNR, i.e. contrast than the polymers relative to the phantom (Table 2). However, the greater contrast of gold was degraded by the artifact to a much greater extent than with the polymer when considering the CNR relative to the ROI, that includes the artifact. SNR was similar for the gold and polmer fiducials relative to the phantom. However, the gold signal was degraded by a more considerable amount by the artifact in the ROI as it obscured the phantom signal. The much larger AI confirms the increased artifact for the gold fiducial relative to the polymer, that degrades the contrast (CNR) and signal (SNR) of the normal tissue phantom immediately surrounding the fiducial. This is consistent with the CT appearance.

**Table 2:** Comparison of the Mean (Standard Deviation, SD) of the Contrast to Noise Ratio (CNR), Signal to Noise Ratio (SNR) and Artifact Index (AI) of the polymer and gold fiducials in CT and MRI imaging. The means (SD) relate to the CT number for CT scans and gray-scale for the MRI. Two comparative Backgrounds were used for CNR and SNR, i.e. distant to the artifact for “Phantom”, and the 50 mm square region of interest (ROI) containing the artifact for the “ROI”.

Statistic: Mean (SD)	Polymer	Gold
**CT**		
CNR (Phantom)	89.4 (25.4)	491.9 (24.7)
CNR (ROI)	54.2 (3)	30.1 (7.9)
SNR (Phantom)	103.5 (3.3)	97 (5.2)
SNR (ROI)	65.6 (15.3)	5.9 (1.3)
AI	13.7 (5.6)	191.6 (41.4)
**MRI**		
CNR (Phantom)	27.3 (5.1)	29.4 (4.4)
CNR (ROI)	21.2 (1.2)	24 (3.5)
SNR (Phantom)	70.2 (9.8)	106.9 (9.1)
SNR (ROI)	52.8 (1.3)	85.4 (12.1)
AI	5.8 (0.9)	4.1 (1)

CNR = Contrast/Noise = (Fiducial mean − Background mean)/(Background SD). SNR = Signal/Noise = (Background mean excluding fiducial)/(Background SD excluding fiducial).

AI = $$\sqrt{({\mathrm{ROI \,SD \,excluding \,fiducial})}^{2}-{\left(\mathrm{Background \,SD \,excluding \,fiducial}\right)}^{2}}$$.

**Figure 4:**
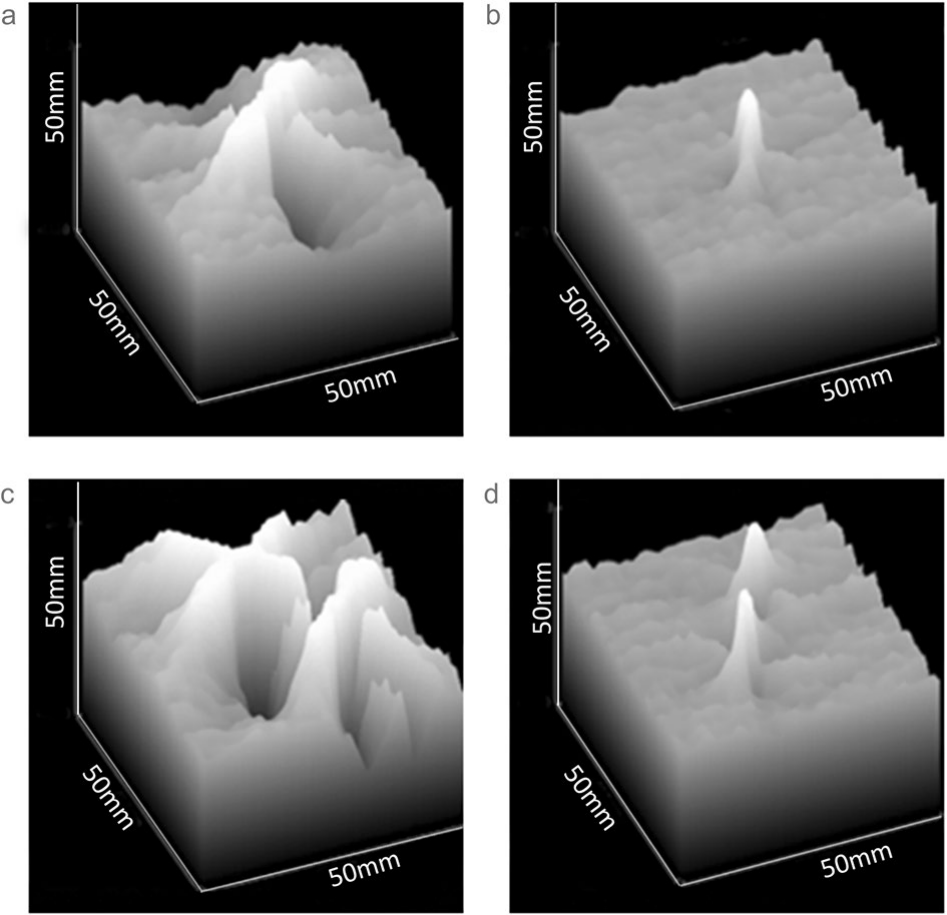
Area gray-scale “terrain” of a 50 × 50 mm square centered on (**a**) Single gold seed, (**b**) Single polymer seed, (**c**) Two adjacent gold seeds, and (**d**) Two adjacent polymer seeds.

Surface area gray-scale profiles illustrated the fiducial’s impact on the surrounding phantom and demonstrated the interaction of multiple fiducials (Fig. 4). The gold fiducial produces a central peak but is surrounded by multiple irregular peaks and troughs of gray-scale, representing the noisy artifacts around the fiducial (Fig. 4a). The polymer fiducial produces a single well-defined peak with little perturbation of the surrounding gentle undulating terrain of the phantom gray-scale (Fig. 4b). This is further exemplified when two fiducials are in close proximity to each other, as would not uncommonly be seen in the clinical situation (Fig. 4c,d).

### MRI scan

On MRI, the visual analysis showed that the gold and polymer fiducials have a similar appearance being hypo-intense and thus dark on T2 weighted imaging relative to the phantom (Fig. 1e,f). A mild uniform hyperintense area around the fiducials was slightly more prominent with the gold fiducials. At one end of the polymer fiducials, a pronounced hyperintense area was due to the wax used to hold the fiducial in the needle. This was not present with the gold fiducial. The wax would usually be absorbed in vivo and thus would not be present on a clinical MRI.

**Figure 5:**
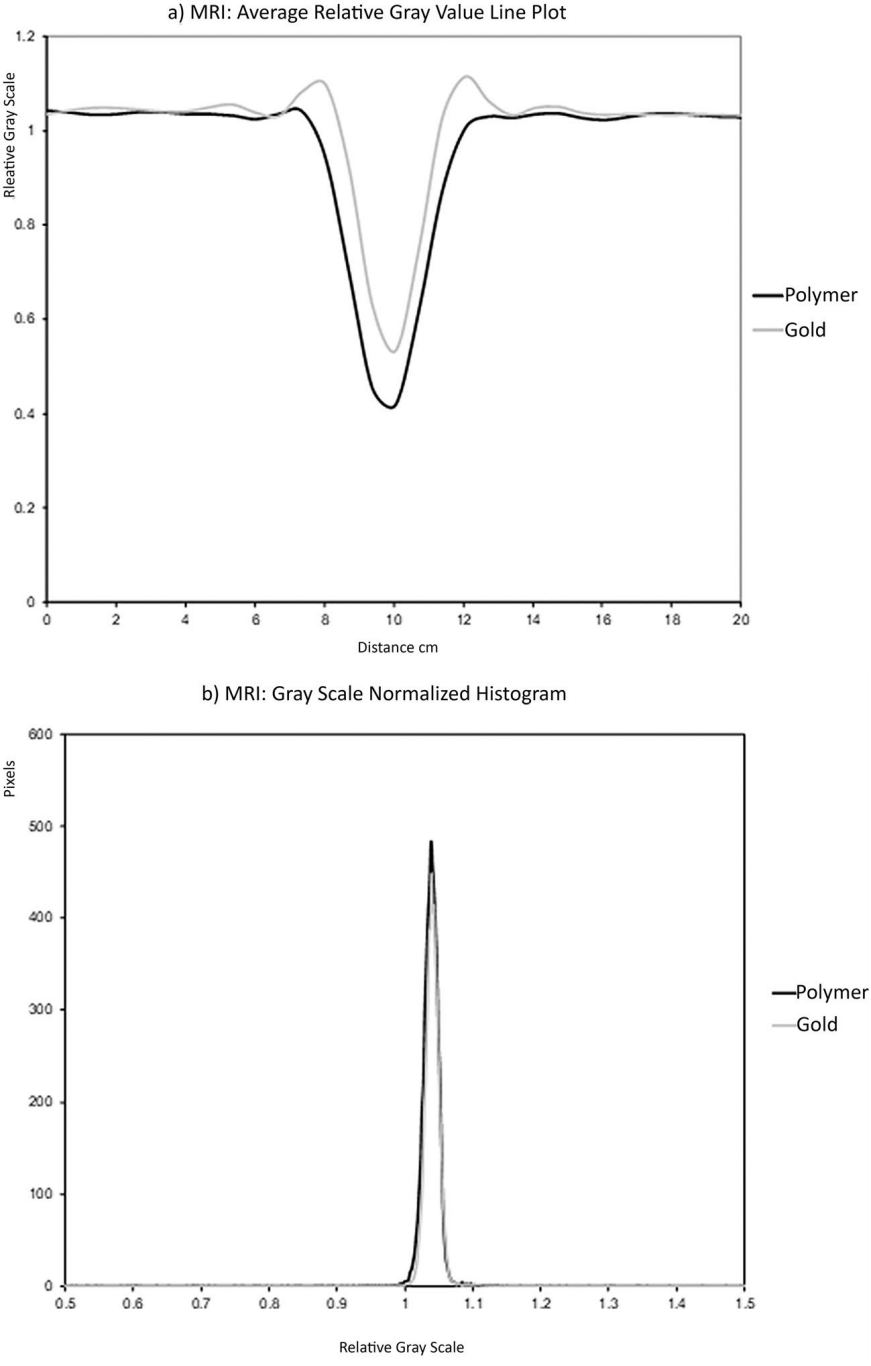
(**a**) line profile of average relative pixel gray values along a 20 mm line centered on each fiducial marker on MRI (**b**) MRI histogram of average relative pixel gray values within a 50 × 50 mm square centered on each fiducial marker, polymer, and gold on MRI.

A 20 mm MR line profile was used with MRI because of the minimal artifacts from either fiducial (Fig. 5a). The gold and polymer plots have a similar appearance. The main differences were that the polymer fiducials had a slightly darker gray-scale trough than the gold fiducials (polymer relative gray-scale value reaching a minimum of 0.4181 compared to 0.5328 for gold fiducials). Notably, there are small increases in the gray-scale on either side of the peak base with the gold fiducials. This represents the small bright or hyperintense hue artifact around the fiducials, particularly the gold fiducials. The polymer fiducials line profile appears slightly wider than the gold but may result from the polymer fiducial being slightly wider in diameter or a slight increase in dark artifact around the polymer fiducial.

The histogram of the MRI images shows that the polymer and gold fiducials plots are similar in appearance (Fig. 5b). The polymer MRI normalized histograms show slightly wider percentile ranges (1st to 99th percentile: 0.9636 to 1.0339) when compared to the gold seeds (0.9748 to 1.0315), and a slightly larger standard deviation about the centralized mean value (0.5050 compared to 0.0455) (Table 1). The surface area profiles of both fiducials further showed the similarity between the two types of fiducials markers in that they produce minimal artifacts.

The gold and polymer fiducials’ MRI CNR values show much smaller differences for MRI than CT relative to both phantom and ROI (Table 2). Gold has a higher SNR than polymer, but the AI is almost equivalent. Thus, while gold has a higher signal, the contrast and artifact are similar to the polymer fiducials and are consistent with the visual appearance.

## Discussion

The increasing precision of prostate radiotherapy requires a fiducial that can be visualized on multi-modality imaging with minimum artifacts. Therefore, to maintain consistency across imaging, a phantom was developed that was both CT and MRI tissue-equivalent. This is the first phantom to our knowledge with such dual properties. This phantom study has shown that polymer fiducials can be well seen on modern three-dimensional imaging, such as MRI and CT. They produce fewer CT artifacts than the benchmark gold fiducials and appear at least equivalent to gold on MRI. This study further reinforces the findings in a recent prostate CT tissue-equivalent phantom study that included polymer fiducials but provided further information by developing a phantom that was both CT and MRI tissue equivalent^14^.

The accuracy of MRI to planning CT co-registration may be improved with polymer fiducials matching as they are visible on CT and MRI. It could also reduce interference from artifacts seen with gold that can conceal crucial soft tissue structures such as the prostate capsule, apex, and dominant intraprostatic lesion (DIL). The accurate co-registration of MRI and CT is essential as MRI defined volumes are significantly smaller than CT because less normal tissue is included in target volumes^15–17^. It improves the contouring uncertainty, particularly at the apex and base, and reduces the inter-observer and intra-observer variation^6,7,18,19^ and the dose to normal tissue^20^. MRI also better defines prostate cancer pathology, such as the DIL, extracapsular extension, and seminal vesicle invasion. Metallic structures, such as gold, cause CT imaging anomalies, including distortion, metal artifacts, and change in target density^21^. The distorted CT image can also result in inaccurate planning and delivery if not accounted for ^21^. Polymer fiducials may minimize this problem.

Polymer fiducials may provide more accurate co-registration of CBCT to planning CT for treatment verification or IGRT. Comparison studies of IGRT/fiducial markers with IMRT versus non-IGRT treatments have shown a decrease in late GI and GU toxicity^2,22,23^. In one study, there was also an improvement in clinical outcome^2^. The toxicity difference can be attributed to the combination of the IMRT technique with a reduced dose to organs-at-risk, daily image guidance, and margin reduction that IGRT with fiducials safely permits.

The study’s limitation is that the phantom was a uniform tissue density and a mobile size to provide an appropriate test environment. However, a human subject will have a greater thickness and heterogeneity, which may interfere with the fiducial visibility. Furthermore, the study did not analyze other fiducials available on the market. There are new fiducial markers that produce minimal distortion with CT imaging. Visicoil uses helical coils of gold to reduce the relative thickness and decrease the equivalent density, thus reducing image artifact^24^. Others use a mixture of low-density biocompatible materials and gold particles^25^. Additional alternatives utilize lower Z radiopaque materials such as stainless steel, titanium^26^, and carbon or ceramic materials^8,10^.

Another limitation is that we did not have access to artifact suppression CT^21^ during the investigation. While artifact suppression CT is becoming more common in clinical practice, it is not widespread in radiotherapy and not available for cone-beam CT. Alternative MRI sequences were not used because of the center’s MRI time limitations. Investigations have shown that other MRI sequences such as T2*2D & T2*3D^27^ and multi-parametric MRI with bTFE (balanced steady-state free precession sequence)^28^ are better at visualizing gold seeds. These sequences are not used commonly in radiotherapy, but their implementation being investigated with the recent advent of MRI simulators and MRI linacs in radiotherapy.

Nevertheless, these phantom results indicate that polymer fiducials may be more appropriate for X-ray-based imaging than the current standard gold marker. They are visualized as a discrete structure on CT and produce little to no image artifact. Given the CT visualization improvements, combined with equivalent MRI visualization and similar size and physical appearance, the polymer fiducial could improve radiotherapy treatment planning and localization. However, they are more expensive than standard gold seeds.

The study was limited to the investigation of CT and MRI simulation images. The rationale was to focus on the major radiotherapy imaging tools and consider polymer fiducials to aid image co-registration. CT based imaging, where the gold metallic artifact is an issue, forms the central part of contemporary radiotherapy in terms of geometry and dosimetry. Whereas MRI has had a secondary function, being co-registered to CT. Its main function has been the identification of prostate anatomy and cancer pathology. However, MRI is becoming increasingly important as the primary modality of simulation and verification with the clinical introduction of MRI linacs^29,30^. While the polymer fiducials have been approved for radiotherapy verification, future patient investigations are warranted to thoroughly test them against the standard gold fiducials across all modern verification modalities, including electronic portal imaging (EPID), cone-beam CT, and MRI. Notably, the polymer fiducials had less contrast than the gold fiducials, as evident from the CNR calculations. While gold fiducials are clearly seen for EPID or orthogonal KV verification, it is yet to be shown whether the polymer fiducials can be visualized with orthogonal matching, particularly lateral planar imaging, where pelvic bones could obscure the lower contrast fiducials.

We have shown that polymer fiducials have good visibility and reduced artifacts compared to gold fiducials. The reduced CT artifact could enhance radiotherapy quality and accuracy by improving the visualization of critical targets such as the prostate apex and allowing for more precise fiducial to fiducial verification, devoid of obscuring artifact. The polymer fiducials may have utility alongside the development of MRI linacs practice delivery protocols. However, despite the encouraging results of this preliminary phantom study, polymer fiducials’ real value and cost-effectiveness in radiotherapy must be validated clinically before they can replace gold fiducial markers. A comparison of polymer and gold fiducials in prostate cancer patients is indicated based on these encouraging results.

## Data Availability

Research data is stored in an institutional repository and will be shared upon request to the corresponding author.
